# Polymer Nanoparticles: Synthesis and Applications

**DOI:** 10.3390/polym14245449

**Published:** 2022-12-13

**Authors:** Suguna Perumal

**Affiliations:** Department of Chemistry, Sejong University, Seoul 143747, Republic of Korea; suguna.perumal@gmail.com

Polymer nanoparticles (PNPs) are generally formed by the spontaneous self-assembly of polymers that vary size from 1 to 1000 nm [1]. Self-assembly of polymers or surfactant-directed polymers forms the PNPs. Self-assembly of polymers available at critical micelle concentration (CMC). CMC is a concentration above which PNPs are formed [2]. Typical PNPs are normal micelles and inverse micelles, as shown in Figure 1. The normal micelle will have a hydrophobic/oil core and hydrophilic/water shell, while the inverse micelle will have a hydrophilic/water core with a hydrophobic/oil shell [3]. In addition to normal or inverse structures, self-assembly nanostructures of PNPs include sphere, tubular, bottle-brush, rod-shaped, and so on [4].

The preparation of PNPs are achieved by solvent evaporation, salting out, nanoprecipitation, desolvation, dialysis, ionic gelation, and spray drying methods [5,6]. Different types of polymers were employed for the preparation of PNPs, which includes natural polymers, for instance, gelatin, alginate, and albumin, and synthetic polymers such as random block copolymer, grafter polymer, block copolymer, and ionic polymers form PNPs [7,8]. PNPs show a wide range of applications which have been extensively employed as biomaterials in recent years because of their characteristic features. This includes biocompatibility, small size, high surface–volume ratio, and tunable surface and structure [1]. In addition to biomaterials applications such as drug delivery, imaging, biosensors, and stimuli-responsive systems, PNPs are used in environmental and agricultural applications [9,10,11,12,13,14,15]. The small size of PNPs permits penetration through capillaries, and thus, they are referred to as nanocarriers. PNPs protect the drug molecules, lead to controlled release, and are thus used in drug delivery and diagnostics applications [10,11]. Due to their high mechanical strength, optical and thermal properties, and conductivity, PNPs are used in imaging, sensors, catalysis, and water treatment applications [12,13,14,15]. 

Thus, this Special Issue was established to cover the exciting studies pertaining to polymeric materials and their applications. Romero et al. [16] reported about Pluronic F-127 stabilized polymeric lipid hybrid nanoparticles (PLHNs). Curcumin drugs, demethoxycurcumin (DMC) and bisdemethoycurcumin (BDM) were loaded in PLHNs. The prepared DMC-loaded PLHNs and BDM-loaded PLHNs were characterized by many techniques. Overall, 88% of DMC and 68% of BDM were released from DMC- and BDM-loaded PLHNs at 180 min. The IC_50_ values for DMC- and BDM-loaded PLHNs were lower than the free ethanolic solutions of DMC and BDM. This confirms the improvement of antioxidant activity using DMC- and BDM-loaded PLHNs particles. Ruiz-Bermejo et al. [17] presented for the first time, the synthesis of submicron particles using diaminomaleonitrile polymers by microwave radiation. The reaction time was varied as follows: 16 min at 170 °C and 3.2 min at 190 °C. The structural, thermal, and electrochemical properties were studied carefully using various techniques. The obtained particles were ~230 nm with a long rice-like shape structure. The prepared polymers exhibited good semiconductor properties and can thus be a potential candidate for soft polymer materials. 

Apart from the polymeric nanoparticles, the metal-incorporated polymer nanoparticles, and the preparation of metal nanoparticles from plant sources are also focused on. Salleh et al. [18] explained the synthesis of silver nanoparticles (AgNPs) using natural pullulan (AgNPs/PL) by the γ-irradiation process. The prepared AgNPs/PL was characterized by UV-Vis spectroscopy, X-ray powder diffraction (XRD), transmission electron microscopy (TEM), and Zeta potential analyses. Further, the AgNPs/PL was analyzed for antimicrobial activity against *Staphylococcus aureus* which showed high antibacterial activity with 11–15 nm as an average diameter of the inhibition zone at higher irradiation doses as 50 kGy. Grape pomace-extracted tannin was used as a reducing and stabilizing agent for AgNPs [19]. The prepared Ta-AgNPs showed a maximum at 420 nm in UV-Vis spectroscopy. The zeta potential measurement value of −28.48 suggests the stability of Ta-AgNPs. The surface morphology studies using TEM showed a size between 15 and 20 nm. Ta-AgNPs exhibited antidiabetic activity inhibition of α-amylase and α-glucosidase with IC_50_ values of 48.5 and 40.0 μg/mL, respectively. In addition, Ta-AgNPs were employed as a potent antioxidant and antibacterial agent. Polymeric nanoparticles were prepared using 2-hydroxyethyl methacrylate as a backbone monomer, ethylene glycol dimethacrylate as a cross-linker, and methacrylic acid as a functional monomer [20]. The prepared polymer was loaded with zinc and calcium nanoparticles, and their antibacterial effect was studied using an in vitro subgingival biofilm model. The prepared polyester-stabilized AgNPs and their antimicrobial performance against *Staphylococcus aureus* and *Escherichia coli* were systematically studied and reported [21]. The extract from the medicinal plant “Thymus serpyllum” was reported as a stabilizing and reducing agent in the preparation of AgNPs [22]. The antidiabetic activity on Streptozotocin-induced diabetic BALB/c mice was reported. Perumal et al. [23] synthesized the water-dispersible graphene composite. The graphene surface was functionalized with zwitterion polymer poly [2-(methacryloyloxy)ethyl]dimethyl-(3-sulfopropyl)ammonium hydroxide and iron oxide nanoparticle (FeNPs). The prepared composites were confirmed from various analyses such as XRD, Raman, SEM, TEM, X-ray photoelectron spectroscopy, and thermogravimetric analysis.

Moreover, Liu et al. [24] has summarized the glass transition temperature (*T*_g_) of poly(lactic-co-glycolic acid) (PLGA) particles and their application towards drug delivery. The change in *T*_g_ of PLGA particles with a change in size, molecular weight, shape, and with ionic liquids was discussed in detail. The *T*_g_ of PLGA showed as an indicator for the controlled drug release. Vinodh et al. [25] reviewed and concisely reported on the polysulfone-based membrane for fuel cell application.

Thus, the articles that are published in this Special Issue will of particular interest for researchers who work with polymer materials. Additionally, these articles will be helpful in the further development of polymer materials for diverse applications.

## Figures and Tables

**Figure 1:**
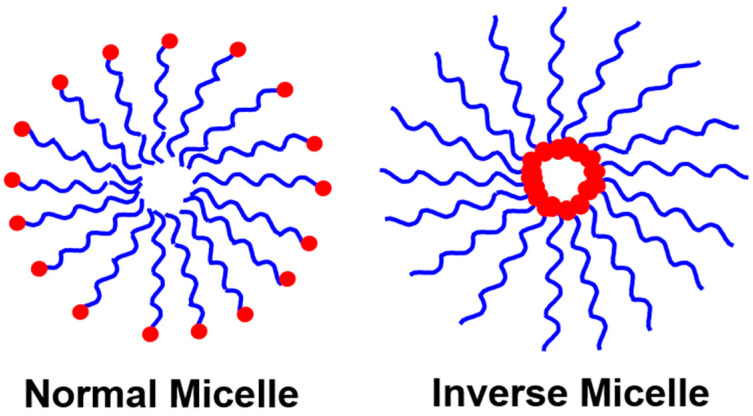
Schematic representation of normal and inverse micelles.
